# Rapid identification of homozygosity and site of wild relative introgressions in wheat through chromosome‐specific KASP genotyping assays

**DOI:** 10.1111/pbi.13241

**Published:** 2019-09-30

**Authors:** Surbhi Grewal, Stella Hubbart‐Edwards, Caiyun Yang, Urmila Devi, Lauren Baker, Jack Heath, Stephen Ashling, Duncan Scholefield, Caroline Howells, Jermaine Yarde, Peter Isaac, Ian P. King, Julie King

**Affiliations:** ^1^ Nottingham BBSRC Wheat Research Centre School of Biosciences University of Nottingham Loughborough Leicestershire UK; ^2^ LGC Biosearch Technologies Hoddesdon Hertfordshire UK; ^3^ IDna Genetics Ltd. Norwich Research Park Norwich UK

**Keywords:** KASP assays, chromosome‐specific, wheat, wild relatives, homozygous, introgressions

## Abstract

For future food security, it is important that wheat, one of the most widely consumed crops in the world, can survive the threat of abiotic and biotic stresses. New genetic variation is currently being introduced into wheat through introgressions from its wild relatives. For trait discovery, it is necessary that each introgression is homozygous and hence stable. Breeding programmes rely on efficient genotyping platforms for marker‐assisted selection (MAS). Recently, single nucleotide polymorphism (SNP)‐based markers have been made available on high‐throughput Axiom^®^ SNP genotyping arrays. However, these arrays are inflexible in their design and sample numbers, making their use unsuitable for long‐term MAS. SNPs can potentially be converted into Kompetitive allele‐specific PCR (KASP™) assays that are comparatively cost‐effective and efficient for low‐density genotyping of introgression lines. However, due to the polyploid nature of wheat, KASP assays for homoeologous SNPs can have difficulty in distinguishing between heterozygous and homozygous hybrid lines in a backcross population. To identify co‐dominant SNPs, that can differentiate between heterozygotes and homozygotes, we PCR‐amplified and sequenced genomic DNA from potential single‐copy regions of the wheat genome and compared them to orthologous copies from different wild relatives. A panel of 620 chromosome‐specific KASP assays have been developed that allow rapid detection of wild relative segments and provide information on their homozygosity and site of introgression in the wheat genome. A set of 90 chromosome‐nonspecific assays was also produced that can be used for genotyping introgression lines. These multipurpose KASP assays represent a powerful tool for wheat breeders worldwide.

## Introduction

Bread wheat (*Triticum aestivum* L.) is one of the most widely grown crops in the world and accounts for almost one‐fifth of the human calorie intake (FAO, 2017). Its allohexaploid (AABBDD; 2n = 6x = 42) genome was derived from the hybridization of diploid *Aegilops tauschii* (DD; 2n = 2x = 14) with tetraploid *Triticum turgidum* ssp. *dicoccoides* (AABB; 2n = 4x = 28) (Dubcovsky and Dvorak, 2007; Matsuoka, 2011). Due to this hybridization event, followed by domestication and inbreeding, genetic variation has reduced in modern cultivated wheat (Haudry *et al*., 2007). However, genetic diversity is crucial if the wheat species is to survive and adapt to the threat of abiotic and biotic stresses. It has been suggested that interspecific crossing of wheat with its wild relatives can enrich wheat's gene pool with novel diversity (Reynolds *et al*., 2011). One strategy, recently called ‘introgressiomics’ (Prohens *et al*., 2017), consists of a whole‐genome introgression approach involving transfer of chromosome segments from the entire genome of a wild relative species into the wheat background, irrespective of any traits that the wild relative might carry and a number of such studies have already been undertaken (Grewal *et al*., 2018a,b; King *et al*., 2017, 2018; Valkoun, 2001). In this prebreeding strategy, the interspecific hybrids are repeatedly backcrossed to the elite wheat parent to reduce the number and size of the introgressed segments and self‐fertilized to obtain stable homozygous introgressions that can be utilized for trait analysis (King *et al*., 2019). Previously, wild relative introgressions were detected using labour‐intensive cytogenetic techniques (Lukaszewski *et al*., 2005). More recently, molecular markers provide high‐throughput and cost‐effective evaluation of introgressions in large numbers of lines (Thomson, 2014).

Some studies have used co‐dominant markers such as simple sequence repeats (SSRs) to detect wild relative introgressions in wheat (Adonina *et al*., 2004; Qi *et al*., 2010; Quarrie *et al*., 2005; Rodríguez‐Suárez *et al*., 2011; Zhao *et al*., 2013). However, with these being cost‐ineffective, laborious and time‐consuming to use, they have limited potential in wheat breeding programmes. Single nucleotide polymorphism (SNP) markers, on the other hand, have now become commonplace in wheat genotyping (Akhunov *et al*., 2009; Bevan and Uauy, 2013; Davey *et al*., 2011) and marker‐assisted selection (MAS). However, in polyploid species such as wheat, the development of SNP markers has been challenging due to the presence of homoeologous and paralogous copies of genes (Edwards *et al*., 2009; Kaur *et al*., 2012) and distinguishing between interspecific SNPs from intergenomic polymorphisms within wheat can be complicated and error‐prone (Akhunov *et al*., 2009). Exome‐based sequencing has provided a huge resource of SNPs between wheat varieties (Allen *et al*., 2013; Winfield *et al*., 2012). Many of these have been developed into high‐density SNP arrays (Allen *et al*., 2017; Rimbert *et al*., 2018; Wang *et al*., 2014; Winfield *et al*., 2016) for high‐throughput genotyping in wheat. An Axiom^®^ Wheat‐Relative SNP Genotyping Array has also been developed and used in studies for the identification and characterization of wild relative introgressions in a wheat background (Grewal *et al*., 2018a,b; King *et al*., 2017, 2018). Although these SNP genotyping platforms can be ultra‐high‐throughput and efficient, their use in crop breeding has been limited because they are inflexible in their design and use (Rasheed *et al*., 2017). This leaves wheat breeders who want to carry out medium‐ to low‐density genotyping on large numbers of plants with very few options.

More recently, the Kompetitive allele‐specific PCR (KASP™) system has been demonstrated to be a more flexible, efficient and cost‐effective system for genotyping in wheat (Allen *et al*., 2013; Neelam *et al*., 2013; Tan *et al*., 2017; Yu *et al*., 2017) and other crop species (Semagn *et al*., 2014; Steele *et al*., 2018; Zhao *et al*., 2017). The KASP system allows (i) conversion of SNPs from fixed chip platforms to a stand‐alone format where hundreds to thousands of samples can be genotyped with relatively fewer markers and (ii) flexibility of customization of the genotyping run with different combinations of SNP markers and sample numbers. But this technology has two major drawbacks for wheat–wild relative genotyping. Firstly, it requires the identification and characterization of interspecific SNPs among an excess of homoeologous and paralogous SNPs. Secondly, as this platform was primarily developed for diploid species, there are problems with the scoring of interspecific SNPs in polyploid heterozygotes, such as segregating backcross populations. For the KASP system to detect a wild relative segment in an allohexaploid wheat background, it has to accurately distinguish between different call ratios (Allen *et al*., 2011, 2013). For example, if the KASP assay is for a SNP, which has three homoeologous copies in wheat, it will be extremely difficult to distinguish between a heterozygous introgression having a call ratio of 5:1 and a homozygous introgression having a call ratio of 4:2, in a self‐fertilized backcross line (Allen *et al*., 2011). In contrast, if the SNP assay is for a SNP which amplifies only a single homoeologous/paralogous copy in wheat (co‐dominant), then this system would be easily capable of differentiating between a heterozygous (call ratio of 1:1) and a homozygous (call ratio of 2:0) introgression in a segregating population.

A recent study successfully converted a panel of PCR markers to KASP™ markers for functional genes in wheat (Rasheed *et al*., 2016). A number of array‐based, putative co‐dominant SNPs have been reported for various wild relatives (Grewal *et al*., 2018a,b; King *et al*., 2017, 2018), which could potentially be converted into KASP™ assays. However, it is difficult to design PCR primers for array‐based probes due to the high level of sequence polymorphism between wheat and its wild relatives. It is also possible that homoeologous sequences that may not have bound to array‐based probes due to sequence divergence could be amplified by the KASP primers. However, careful primer design can lead to the successful amplification of just one homoeologue/paralog. Moreover, targeting single‐copy regions of the wheat genome for SNPs could be a more fruitful strategy since these sequences, by definition, will not have homoeologous copies and, thus, should not suffer from the interference usually encountered.

In this study, we have exploited chromosome‐specific sequences in wheat, that is sequences that are found only on a particular chromosome of wheat, for SNPs with wild relative species. Some of these SNPs were subsequently converted to KASP assays, and where a target SNP sequence was not chromosome‐specific, that is having other homoeologous copies, the KASP assays were designed to potentially amplify only the target subgenome of wheat. This work has resulted in 620 chromosome‐specific co‐dominant KASP assays, evenly spread across the hexaploid wheat genome. These assays will allow rapid identification of homozygosity of wild relative introgressions and their site of recombination within wheat, in segregating populations. In addition, a set of 90 chromosome‐nonspecific KASP assays is also reported that are useful for genotyping lines for the presence of wild relative segments. Validation was carried out through genotyping backcross populations of these wild relative species and genomic *in situ* hybridization (GISH). These KASP assays are valuable tools in wheat–wild relative introgression studies and are predicted to be useful for the detection of many other wheat wild relatives that will be of considerable interest to the wheat research community.

## Results

### SNP discovery

A BLASTN search of all the 36 711 SNP‐containing probe sequences on the Axiom^®^ Wheat‐Relative Genotyping Array against the wheat reference sequence, IWGSC CSS v3 (IWGSC *et al*., 2014), resulted in 2716 probes that had a BLAST hit to only 1 contig (Table 1). From these 2716 target SNP‐containing probe sequences, it was possible to design primers for 2170 from the flanking 500‐bp genomic sequence. Genomic DNA from two wheat varieties, Paragon and Chinese Spring, along with ten wild relatives of wheat, *Amblyopyrum muticum*,* Thinopyrum bessarabicum*,* Thinopyrum intermedium*,* Thinopyrum elongatum*,* Thinopyrum ponticum, Aegilops speltoides*,* Aegilops caudata*,* Triticum urartu*,* Triticum timopheevii* and *Secale cereale*, were used to test the primers for PCR amplification. 1721 primer pairs were successful in amplifying at least one wheat variety and one wild relative species (Table 1). The PCR amplification resulted in 13 731 PCR products that were sent for sequencing. Of these, 61.67% of samples were successfully sequenced. Table 1 shows the distribution of the number of primer pairs designed and samples sequenced across the 21 chromosomes of wheat.

**Table 1:** Distribution of the number of probes on the Axiom^®^ Wheat‐Relative Genotyping Array with BLASTN hit to a single contig in the wheat genome, the primers designed from these probes, PCR products sent for sequencing, the SNPs discovered and selected for KASP assay design

Wheat chromosome	Probes on WRGA† chip with blast to 1 contig‡	Primer pairs designed	Primers worked	PCR products sent for sequencing	Successfully sequenced samples	Successfully sequenced samples (%)	SNPs discovered (across all 10 species)	SNPs sent for KASP assay design
1A	83	63	54	416	276	66.35	93	45
1B	141	99	78	507	312	61.54	135	56
1D	144	99	83	608	334	54.93	154	46
2A	112	91	79	540	396	73.33	119	48
2B	159	132	99	605	442	73.06	141	54
2D	130	111	93	633	447	70.62	126	43
3A	85	86	86	747	278	37.22	117	45
3B	294	266	192	1469	1009	68.69	168	53
3D	79	76	62	469	296	63.11	126	45
4A	95	77	75	833	447	53.66	96	46
4B	88	73	57	415	224	53.98	65	35
4D	73	64	67	586	329	56.14	90	43
5A	68	55	46	382	262	68.59	78	49
5B	167	122	112	833	530	63.63	115	56
5D	155	110	97	729	426	58.44	116	47
6A	102	97	83	642	501	78.04	113	45
6B	92	84	72	531	348	65.54	107	53
6D	140	128	119	897	604	67.34	125	49
7A	90	60	49	403	220	54.59	73	44
7B	140	89	74	521	301	57.77	111	47
7D	279	188	44	965	469	48.60	106	51
Total	2716	2170	1721	13 731	8451	61.67	2374	1000

^†^Axiom^®^ Wild Relative Genotyping Array.

^‡^BLASTN against IWGSC CSS v3.

Orthologous sequences from the wild relative species and at least one wheat variety were aligned to identify putative interspecific SNPs. A SNP was given preference whether it was common between multiple wild relatives. A maximum of one SNP/wild relative species from a primer pair was selected to maximize genome coverage. In total, 2374 putative SNPs, from 8451 sequences, were obtained across all ten wild relative species (Table 1) and their distribution across the wild relative species and the wheat chromosomes is detailed in Table S1. The highest number of SNPs was obtained for *Am. muticum* and *Th. bessarabicum* with 458 each, while the least number of SNPs was 248 and 254, obtained for *Th. ponticum* and *T. urartu*, respectively.

### Primer design for chromosome‐specific assays

The sequence flanking the target SNP was used in a second BLASTN search against the most recent wheat genome sequence, IWGSC RefSeq v1 (IWGSC *et al*., 2018). This additional BLAST search was added to confirm that there were no other homoeologous copies of the target SNP sequences (the improved high‐quality reference assembly for wheat had become available after SNP discovery had been completed). This BLASTN search revealed that only 433 of the 2374 (18.2%) SNP‐containing sequences had a single‐copy in the wheat genome. The remaining sequences had at least one homoeologue, with 67% of the sequences having a homoeologous copy on all 3 subgenomes of wheat (Table S2). The results also showed that 57.5% of the sequences with more than one copy in wheat had homoeologous SNPs; that is, the target SNP was polymorphic between the homoeologues in wheat.

**Figure 1:**
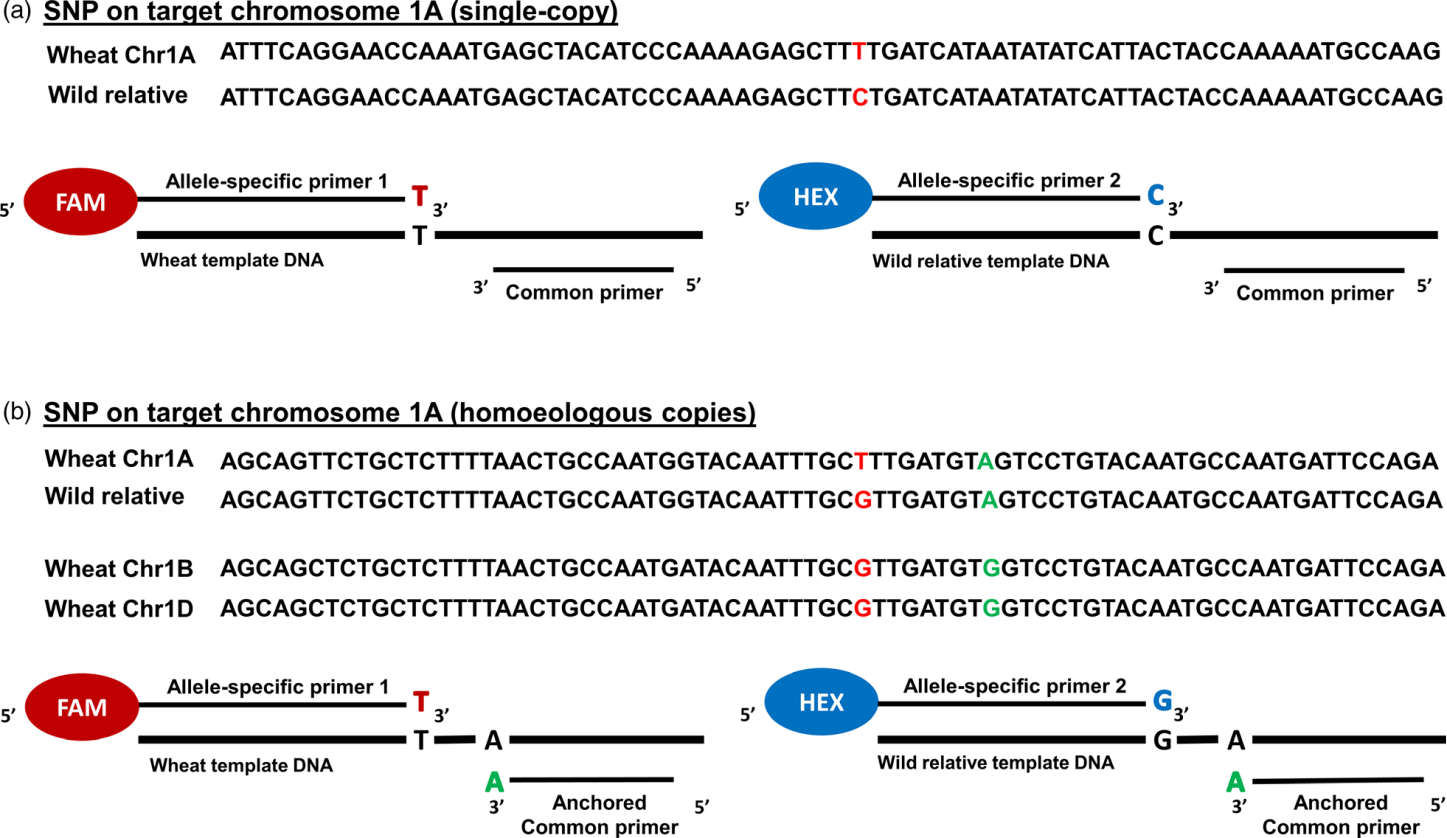
Components of a KASP assay when the target SNP is in a single‐copy region of the wheat genome versus when there are homoeologous copies. (a) For a target single‐copy SNP T/C, on wheat chromosome 1A, the KASP assay mix contains two allele‐specific forward primers and one common reverse primer. (b) For a target SNP T/G, on wheat chromosome 1A having homoeologous copies on chromosomes 1B and 1D, the KASP assay mix contains two allele‐specific forward primers and one common reverse primer with its 3′ end anchored to a base, which is unique to the target subgenome in wheat and is absent in the homoeologous copies. The anchored base is also present in the wild relative sequence. The allele‐specific primers each harbour a unique tail sequence that corresponds with a universal FRET (fluorescence resonant energy transfer) cassette; one labelled with FAM™ dye and the other with HEX™ dye.

Ideally, once a SNP was identified as having flanking sequence suitable for primer annealing, an allele‐specific KASP assay could be designed (Figure 1a). However, in cases where there were homoeologous copies of target SNP sequences, primer optimization was required so that the KASP™ assays would be specific to a particular chromosome in wheat. Thus, a unique base(s) in the flanking sequence of the target subgenome was identified, that is a base(s) that was specific to one homoeologue, but also present in the orthologous wild relative sequence. This single unique base was incorporated into the common primer during the KASP™ assay design to obtain target amplification specificity, also known as primer ‘anchoring’ as shown in Figure 1b. Where such unique bases were identified in the target SNP's flanking sequence, the SNP was categorized as potentially chromosome‐specific. If no specific allele was identified in the target subgenome, the SNP was characterized as chromosome‐nonspecific. Of the 1941 sequences that had more than one copy in wheat, 1488 (76.7%) putative SNPs had the potential for chromosome‐specific assays to be designed, while 453 (23.3%) putative SNPs could only be selected as chromosome‐nonspecific assays (Table S2). Where the target SNP was homoeologous, that is polymorphic within wheat, it was selected only if the assay designed for it was potentially chromosome‐specific (Figure 1b).

### SNP validation and characterization

A subset of 1000 putative SNPs was selected for validation using the KASP™ genotyping platform (Tables 1 and S3) of which 864 were potentially chromosome‐specific and selected to be evenly distributed across the wheat chromosomes. To fill in the gaps, 136 potentially chromosome‐nonspecific SNPs were selected to make up the total to 1000 SNPs. The target SNP sequences, along with any annotations for chromosome specificity, were sent to LGC Genomics for KASP™ assay design. The SNP validation, also performed by LGC Genomics, was done through genotyping various hexaploid and tetraploid wheats, different accessions of ten wild relative species (Table S4), all the Chinese Spring nullisomic–tetrasomic lines plus screening segregating lines (BC_n_F_n_) from the backcrossed populations of the ten wild relatives (Table S5).

For the purpose of this study, SNPs were required between four hexaploid wheat varieties, Paragon, Chinese Spring, Pavon 76 and Highbury, used in the backcrossing programme and various wild relative species. Thus, the initial validation of the KASP markers was based on the genotyping of these four wheats. Of the 1000 putative interspecific SNPs, 710 were polymorphic (between the four wheat varieties and at least one wild relative species), 17 were polymorphic within wheat itself (polymorphism between the homoeologous copies), 3 were polymorphic between the four wheat varieties, and 270 failed to generate a useful amplification signal. Primers were not redesigned when amplification failed. It was noted that of the failed assays, 141 failed to amplify the target wild relative accessions; that is, they only worked for the four wheat varieties or were monomorphic with nontarget wild relatives. To investigate the allelic status of these KASP assays in other wheats, they were used to genotype an additional 12 hexaploid and 15 tetraploid wheat lines (Table S4). Of the 710 KASP markers found to be polymorphic between the four wheats used in this study and the accessions of ten wild relatives, 622 (87.6%) were monomorphic for the wheat allele across all other wheats tested. The genotypes obtained for all the parental and nullisomic–tetrasomic lines are provided in Data S1 including the additional wheat varieties used to validate the marker set.

Since the aim of this work was to produce KASP markers useful for marker‐assisted selection, all the KASP assays were also used to genotype 4666 lines from various segregating and self‐fertilizing backcrossed populations between the wild relatives and the four wheats (Table S5). Introgression lines from each of the ten wild relative species, previously genotyped on the Axiom^®^ Wheat‐Relative Genotyping Array and known to be carrying segments from every linkage group of an individual wild relative species, were included in the genotyping set to ensure that significant proportions of the wild relative genomes were being represented in the KASP marker set.

**Figure 2:**
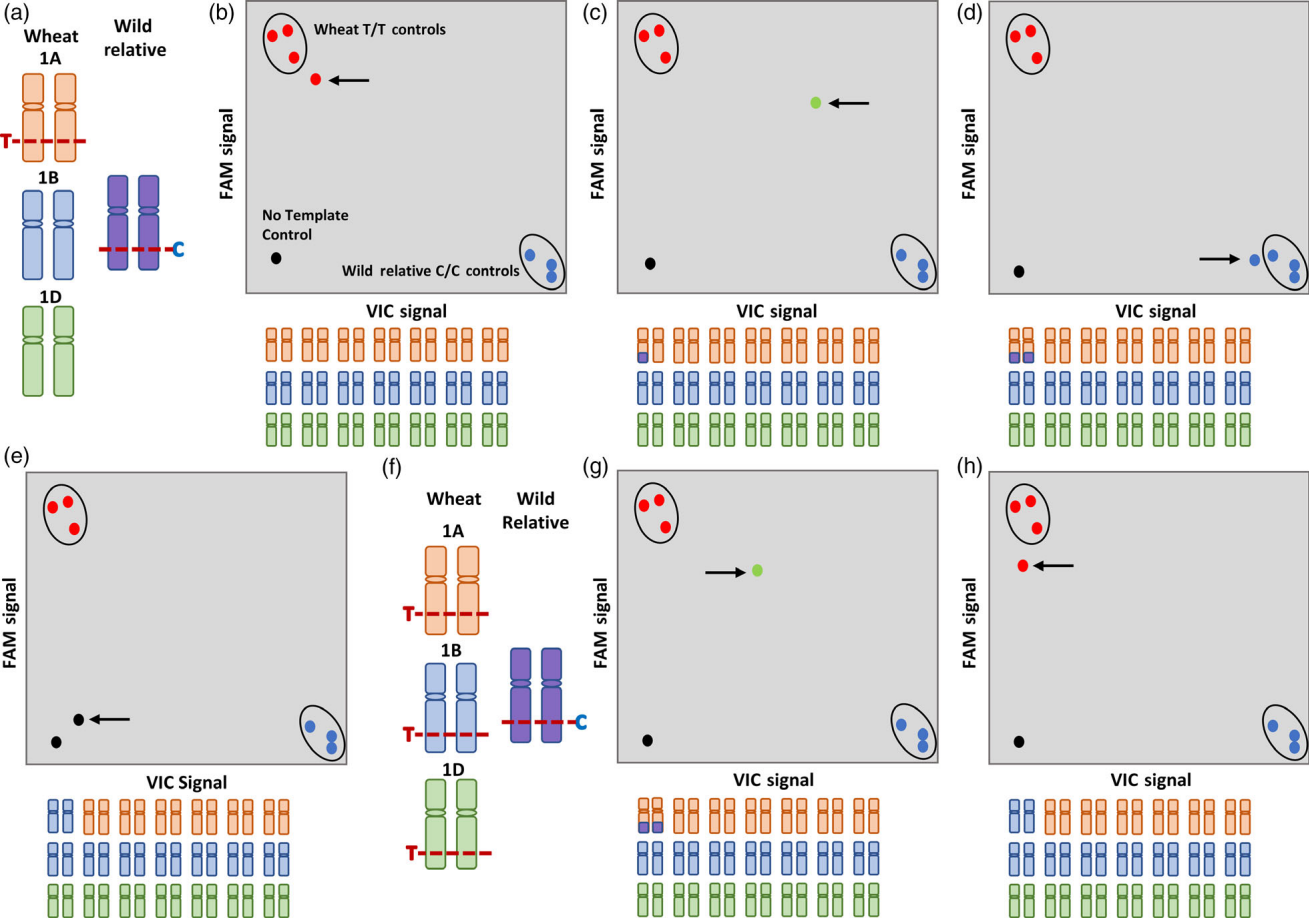
Illustration of genotyping when using chromosome‐specific and chromosome‐nonspecific KASP assays. (a) A chromosome‐specific SNP T/C, on wheat chromosome 1A, used for KASP assay design and genotyping of (b) a line with no wild relative introgression shows a homozygous wheat call (red circle indicated by arrow), (c) a line with a heterozygous introgression shows a heterozygous call (green circle indicated by an arrow), (d) a line with a homozygous introgression shows a homozygous wild relative call (blue circle indicated by an arrow), and (e) a nullisomic–tetrasomic N1AT1D line shows a no call (black circle indicated by an arrow). (f) A chromosome‐nonspecific SNP T/C, having homoeologous copies on wheat chromosomes 1A, 1B and 1D, used for KASP assay design and genotyping of (g) a line with a homozygous introgression shows a heterozygous call (green circle indicated by an arrow), and (h) a nullisomic N1AT1D line shows a homozygous wheat call (red circle indicated by an arrow). In all scenarios, the wheat positive controls are genotyped as T/T (red circles), the wild relative positive controls are genotyped as C/C (blue circles), and the no template control is genotyped as no call (black circle).

Figures 2a‐j depict how genotyping with a chromosome‐specific and chromosome‐nonspecific KASP assay worked. In Figure 2a, the target SNP on chromosome 1A is potentially chromosome‐specific (either because the SNP was only present on a single locus in wheat or the primer design was optimized with primer anchoring as shown in Figure 1b) where the wheat allele is T/T and the wild relative allele is C/C. Screening a line having no wild relative introgression with this assay resulted in a wheat call (T/T; Figure 2b). Screening introgression lines with this assay resulted in three separate clusters for homozygote and heterozygote individuals. When a line had a heterozygous wild relative introgression, this KASP assay gave a heterozygous call (C/T; Figure 2c), but if a line had a homozygous wild relative introgression, the assay resulted in the wild relative call (C/C; Figure 2d). The chromosome specificity was validated when the KASP assay was used to genotype the nullisomic–tetrasomic line (N1AT1B) and resulted in a null call as shown in Figure 2e. Screening the same population with chromosome‐nonspecific SNP assays produced a more scattered cluster where homozygous and heterozygous loci were indistinguishable. For example in Figure 2f, a chromosome‐nonspecific KASP assay for a nonhomoeologous SNP (not polymorphic between the homoeologues in wheat) produced a heterozygous call (C/T; Figure 2g) even if the line had a homozygous wild relative introgression and resulted in a wheat call (T/T; Figure 2h) in the corresponding nullisomic–tetrasomic line N1AT1B (due to the presence of the allele on chromosomes 1B and 1D in both cases). Among the 710 validated polymorphic markers, 620 (87%) were chromosome‐specific in wheat capable of distinguishing between homozygous and heterozygous lines (Table 2), while the remaining 90 KASP™ assays were chromosome‐nonspecific; that is, the SNP was present on more than one homoeologue but not polymorphic between them.

**Table 2:** Distribution of the number of chromosome‐specific KASP assays validated as diagnostic for each of the ten wild relative species

Wheat chromosome	*Th. bessarabicum*	*Ae. caudata*	*Th. elongatum*	*Th. intermedium*	*Am. muticum*	*Th. ponticum*	*S. cereale*	*Ae. speltoides*	*T. timopheevii*	*T. urartu*	Unique assays
1A	10	10	12	14	5	13	4	5	11	10	29
1B	8	10	17	16	10	20	14	15	15	1	36
1D	9	11	18	19	8	19	4	5	7	2	30
2A	8	8	15	14	11	16	7	2	16	16	33
2B	14	11	21	19	13	24	10	20	25	3	41
2D	10	8	15	19	11	16	3	6	9	3	28
3A	4	3	8	11	3	10	7	3	10	12	27
3B	10	11	19	18	10	17	6	14	18	3	36
3D	6	8	14	12	5	12	7	4	4	2	22
4A	7	8	18	18	5	20	9	8	12	12	31
4B	3	6	9	11	11	9	3	11	14	0	25
4D	4	6	18	18	7	13	5	5	5	2	26
5A	8	10	14	14	2	14	10	4	11	11	28
5B	10	14	15	20	6	16	9	17	18	2	37
5D	11	12	18	16	13	19	4	6	10	5	31
6A	10	4	12	15	6	13	5	4	10	10	28
6B	6	7	18	11	7	14	4	11	16	3	31
6D	8	5	8	10	10	9	6	2	7	4	19
7A	5	4	10	7	6	9	3	1	11	9	21
7B	8	4	15	20	11	18	6	9	10	1	32
7D	7	13	9	19	9	13	5	2	10	3	29
Total	166	173	303	322	169	314	131	154	249	114	620

Since many of the chromosome‐specific assays on a chromosome are diagnostic for more than one wild relative species, the last column indicates the number of unique assays that were validated for each chromosome.

**Figure 3:**
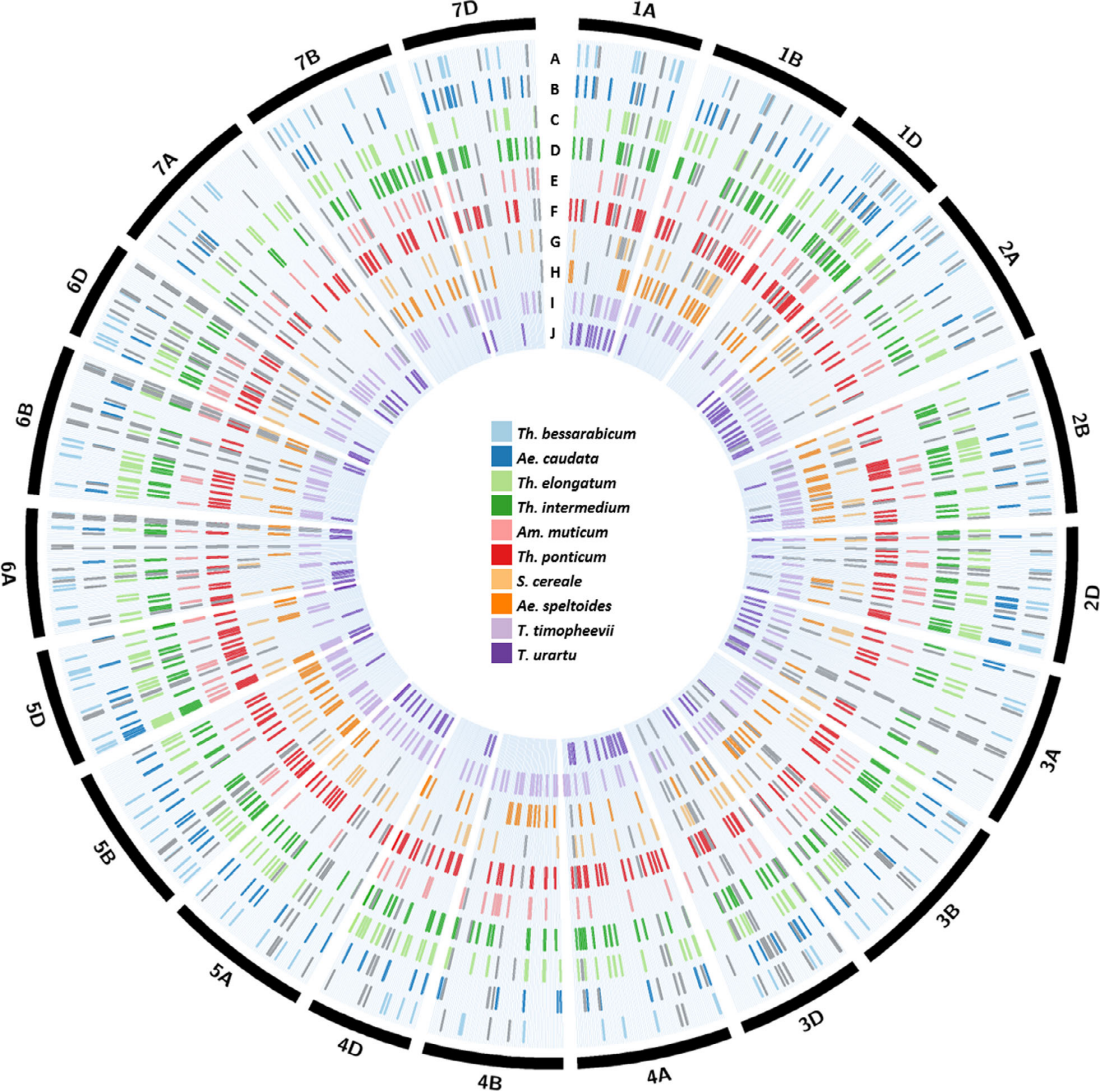
Circular representation of the physical distribution of the chromosome‐specific and nonspecific SNPs, for ten wild relative species, across all wheat chromosomes. A coloured line in each ring represents the physical position, on the wheat chromosome (obtained after BLASTN analysis against the IWGSC RefSeqv1 assembly of the wheat genome; IWGSC *et al*., 2018), of a SNP polymorphic between wheat and (a) *Th. bessarabicum*, (b) *Ae. caudata*, (c) *Th. elongatum*, (d) *Th. intermedium*, (e) *Am. muticum*, (f) *Th. ponticum*, (g) *S. cereal*e, (h) *Ae. speltoides*, (i) *T. timopheevii* and (j) *T. urartu*. SNPs for chromosome‐specific KASP assays are shown in the colour designated to the wild relative species while SNPs for chromosome‐nonspecific assays are shown in grey. The latter are marked for each homoeologous copy in the wheat genome.

Some assays were validated for species in which the SNP had not been detected during SNP discovery. For example, *Th. intermedium* was found to have 322 working chromosome‐specific assays (Table 2) although only 255 SNPs were selected for assay design (Table S3). *Triticum urartu* had the least number of chromosome‐specific assays polymorphic with wheat with 114 spread across the wheat genome and none polymorphic with chromosome 4B (Table 2). Many of the KASP™ assays were diagnostic for more than one wild relative species. Table S6 shows the number of chromosome‐specific assays common between the different wild relatives. The various Thinopyrum species had more assays common between them than with other wild relative species indicating sequence conservation within the Thinopyrum genus. Data S2 shows which wild relative species, each of the 710 validated KASP assays are diagnostic for. More than half of the assays (370 assays) were polymorphic between wheat and at least 3 different wild relative species. The physical location of all the polymorphic SNPs, chromosome‐specific and nonspecific, and their distribution in the wheat genome is represented in Figure 3.

BLASTN analysis showed that 368 of the 620 (62%) chromosome‐specific assays were from single‐copy regions of the wheat genome (Data S3), while the rest had at least one other homoeologous copy. However, due to primer anchoring, the latter only amplified the target chromosomes and were thus classified as chromosome‐specific. A BLASTX search of the chromosome‐specific SNP‐containing sequences against the annotated wheat reference sequence Refseq v1 showed that 275 KASP assays were in protein‐coding regions. Of these, 145 (52%) loci were in single‐copy regions and the remaining 130 had more than one homoeologue in wheat (Data S3). The BLASTN results of the chromosome‐nonspecific assays are shown in Data S4.

### Genotyping with chromosome‐specific markers and validation by genomic *in situ* hybridization (GISH)

In addition to the wheat varieties and the wild relative accessions, the KASP markers were also used to genotype a segregating population derived from each of the ten wild relatives (Table S5). Each wild relative species had a subset of chromosome‐specific markers validated to be polymorphic with wheat (Table 2). Genotyping with these chromosome‐specific markers allowed differentiation of homozygous lines from heterozygous lines in the segregating populations and rapid identification of the wheat chromosome that had recombined with the wild relative segment.

The chromosome‐specific markers, from homoeologous chromosomes in wheat can, collectively, detect the presence of an orthologous wild relative chromosome segment. The genotyping data show that the markers on each of the three subgenomes for a homoeologous group give a heterozygous call when a single wild relative segment from an orthologous group is present. However, if the orthologous segment is homozygous in the introgression lines, then the chromosome‐specific markers on the wheat chromosome involved in the recombination event give a homozygous call (due to the absence of both copies of the wheat allele), while the markers on the other two subgenomes give a heterozygous call.

**Figure 4:**
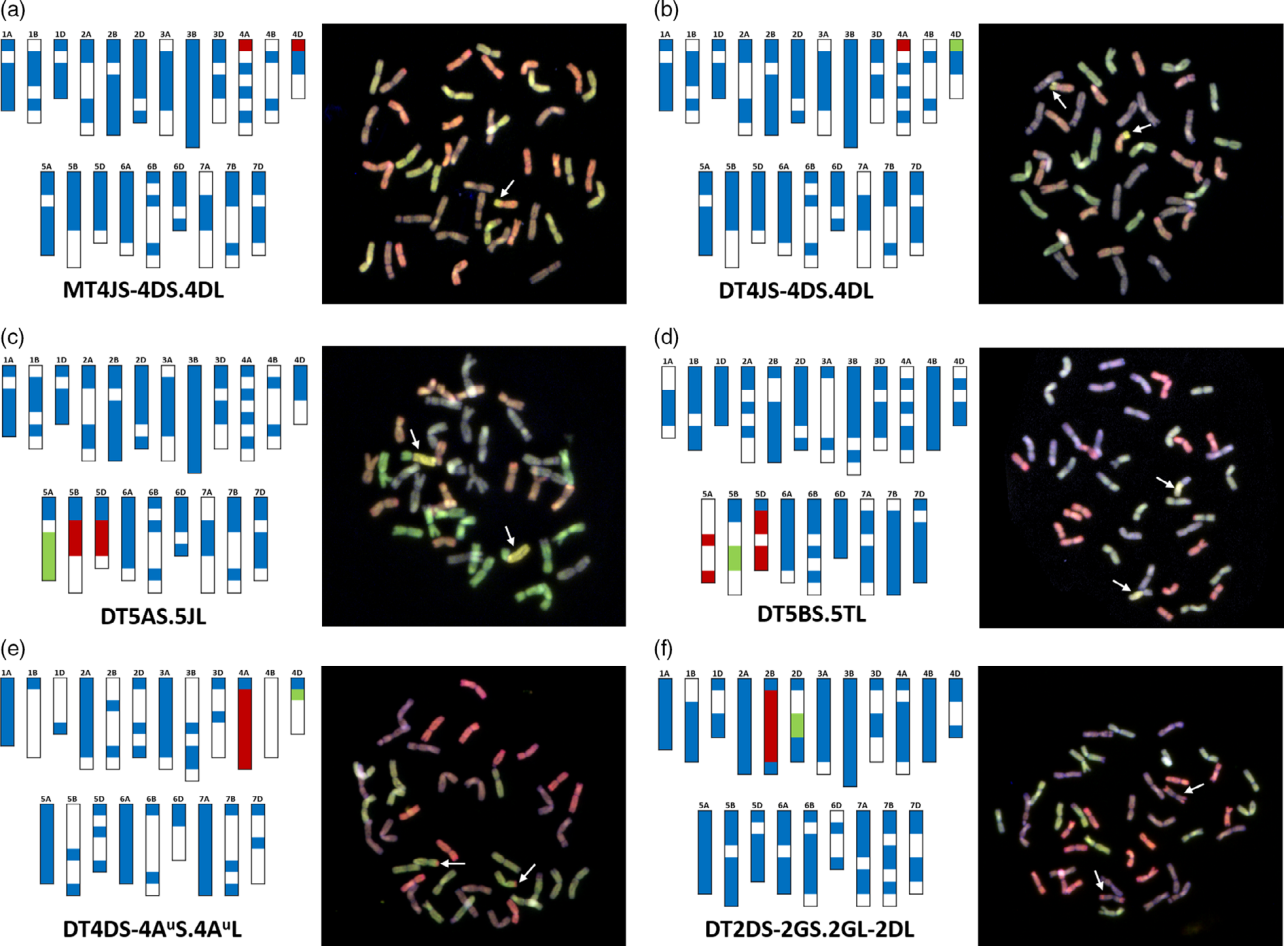
Molecular characterization of wheat–wild relative introgression lines using chromosome‐specific KASP assays and multicolour Genomic *in situ* hybridization (mcGISH). Genotyping data (left), using chromosome‐specific KASP assays, and mcGISH analysis (right) of wheat lines carrying (a) a heterozygous segment from chromosome 4JS of *Th. bessarabicum*, (b) a homozygous segment from chromosome 4JS of *Th. bessarabicum*, (c) a homozygous segment from chromosome 5JL of *Th. bessarabicum*, (d) a homozygous segment from chromosome 5TL of *Am. muticum*, (e) a large homozygous segment from chromosome 4A^u^ of *T. urartu* and (f) a homozygous segment from chromosome 2G of *T. timopheevii*. In the genotyping data, all heterozygous calls are shown in red, homozygous wild relative calls in green and homozygous wheat calls in blue. White spaces indicate where there are no chromosome‐specific KASP assays polymorphic between wheat and the wild relative species. Each wild relative has a species‐specific set of chromosome‐specific KASP assays. For the mcGISH, genomic DNA of *T. urartu* (A genome; green), *Ae. speltoides* (B genome; bluish purple) and *Ae. tauschii* (D genome; red) along with either *Th. bessarabicum* (J genome; yellow) or *Am. muticum* (T genome; yellow) were used as probes. Wild relative segments are indicated by white arrows in the mcGISH images.

Figure 4a–f shows the characterization of introgression lines represented by the genotyping data from chromosome‐specific markers alongside multicolour GISH (mcGISH) analysis of the root metaphase spreads of these lines. The distribution of chromosome‐specific KASP markers, used for genotyping a wild relative species, along the 21 chromosomes of wheat is indicated by coloured regions in the bar diagrams, whereas chromosomal regions lacking the presence of chromosome‐specific markers for that species are indicated by white spaces. Figure 4a–b shows the genotyping of two sister lines containing a segment(s) of chromosome 4JS of *Th. bessarabicum*. The introgression line in Figure 4a is heterozygous for chromosome 4JS as indicated by the presence of heterozygous calls (red regions) for diagnostic chromosome‐specific markers on chromosomes 4AS and 4DS in the genotyping data (the blue regions on all the chromosomes represent markers genotyped as wheat alleles only) and validated by the presence of a single wheat‐*Th. bessarabicum* recombinant chromosome in the mcGISH analysis. Chromosome 4B did not have any chromosome‐specific markers polymorphic with *Th. bessarabicum* in the distal end of the short arm as indicated by a white space. Figure 4b, however, shows homozygous calls (green region) on chromosome 4D (alongside the heterozygous calls on chromosome 4A), indicating that the 4JS segment had recombined with chromosome 4DS of wheat and was homozygous in the line. This was validated by mcGISH that showed the presence of a homozygous chromosome T4JS‐4DS.4DL. Figure 4c shows the genotyping of another wheat‐*Th. bessarabicum* line where the markers indicate the presence of a homozygous group 5 segment, that is chromosome 5J, due to the presence of homozygous calls (in green) on chromosome 5A (alongside heterozygous calls (in red) on chromosomes 5B and 5D). The mcGISH analysis confirmed the presence of homozygous segment T5AS.5JL.

The rapid detection of homozygosity and site of introgression using these chromosome‐specific markers was also obtained in other wheat–wild relative introgression lines as shown in Figure 4d–f. The chromosome‐specific markers were able to detect the homozygous presence of a wheat‐*Am. muticum* recombinant chromosome T5BS.5TL, which was subsequently validated by mcGISH (Figure 4d). The markers also detected *T. urartu* in a wheat background. Figure 4e shows the presence of a large *T. urartu* segment from chromosome 4A^u^ (red heterozygous calls on 4A) with markers, indicating the homozygous segment had recombined with chromosome 4D of wheat (green region). Assuming most *T. urartu* chromosome segments recombine with the A genome of wheat (since *T. urartu* is the progenitor of the A genome of wheat), mcGISH is usually unsuitable for characterizing wheat‐*T. urartu* lines as the genomic probe used to detect *T. urartu* segments cannot differentiate between the *T. urartu* genome and the A genome of wheat. However, since the markers indicated that the *T. urartu* segment had introgressed into the D genome of wheat, mcGISH analysis could validate the presence of this segment as homozygous (Figure 4e). The presence of *T. timopheevii* in wheat was also easily detected by the markers as shown in Figure 4f where a homozygous interstitial segment from *T. timopheevii* chromosome 2G is shown to have recombined with chromosome 2D of wheat. With *T. timopheevii* being a tetraploid (2n = 4x = 28; A^t^A^t^GG), the KASP markers are not only chromosome‐specific in wheat but also for the subgenomes of *T. timopheevii*. Thus, chromosome‐specific markers for the A and B genomes of wheat detect the presence of the A^t^ and G genomes of *T. timopheevii,* respectively. Due to the fact that there is no equivalent of the D genome in *T. timopheevii*, the chromosome‐specific markers on the D genome could be polymorphic randomly with either the A^t^ or the G genomes of *T. timopheevii*. In the case of the introgression line shown in Figure 4f, the markers are detecting the presence of a segment of 2G via heterozygous calls on chromosome 2B (in red) and its presence as a homozygous introgression, in chromosome 2D, due to homozygous calls on chromosome 2D (in green). As with *T. urartu*, mcGISH does not usually work as a detection tool for introgressions from *T. timopheevii* in wheat. However, the detection of *T. timopheevii* is possible via mcGISH, when the markers indicate that the A^t^ genome has recombined with either the B or the D genome of wheat and/or the G genome has recombined with either the A or the D genome of wheat as shown in Figure 4f.

## Discussion

The Axiom^®^ Wheat‐Relative Genotyping Array has been used to genotype various wheat–wild relative introgression populations (Cseh *et al*., 2019; Grewal *et al*., 2018a,b; King *et al*., 2017, 2018). To cost‐effectively genotype the self‐fertilized progenies of these introgression lines, while maintaining high‐throughput scale and flexibility, it was necessary to change the format of genotyping from the Axiom^®^ array to KASP assays. However, initial work to directly convert target SNP sequences into KASP assays was unsuccessful since most of the assays were detecting polymorphic homoeologous loci in wheat (data not shown). This could be likely due to the presence of homoeologous sequences that have diverged in sequence enough for probe annealing to be specific to the target subgenome during array‐type genotyping while allowing amplification of homoeologous sequences by the KASP primers (possibly designed on conserved regions of the sequence). To avoid this, we focused on SNP‐containing probes on the array that potentially had a single‐copy in the wheat genome.

### SNP discovery

At the time of the initial BLASTN search, the assembly used for the analysis was the IWGSC Chinese Spring CSS v3 (IWGSC *et al*., 2014). Approximately 7.4% of the probes on the array were identified as potentially being in single‐copy regions in wheat. Primers were designed to amplify these regions to obtain more information on the extended flanking sequences of the SNPs. Approximately 80% of the identified probes had primers designed from their flanking sequence since not all primer sequence combinations complied with optimum design parameters. In order to find SNPs between wheat and ten wheat relatives (currently under study at the Nottingham BBSRC Wheat Research Centre), it was necessary that the primers amplified at least one of the two wheat varieties used for PCR, along with one or more of the ten wild relative species. From the 2170 primer pairs designed, 79% were successful at such amplification and the resulting PCR products sequenced. A second PCR amplification attempt was made for every failed primer, but it is possible that due to suboptimal conditions some chromosomes were more successful at amplification than others. Multiple sequence alignment of wheat and its wild relatives yielded 2374 putative SNPs from 8451 sequences spread across the 21 chromosomes of wheat (Tables 1 and S1).

### Primer design for subgenome‐specific assays

To verify that SNPs obtained after PCRs and sequence analysis were in single‐copy regions of the wheat genome, a second BLASTN search was conducted using the sequenced amplicons against the new IWGSC wheat genome sequence RefSeq v1 (IWGSC *et al*., 2018). The results showed that less than one‐fifth of the sequences belonged to single‐copy regions, with most having 3 homoeologous copies in wheat (Table S2). This is potentially due to the difference between the quality of the two genome assemblies used for the BLASTN searches, since the key distinguishing feature of the IWGSC RefSeq v1 is that it is an assembly of long reads, with 90% of the genome represented in superscaffolds larger than 4.1 Mb (IWGSC *et al*., 2018), making it a more reliable, high‐quality reference assembly for wheat.

It was possible to annotate sequences to allow ‘anchoring’ of the common primer to a subgenome‐specific base, thereby optimizing the primer design to produce target‐specific KASP assays as shown in Figure 1b. Previous studies have used this technique successfully to design chromosome‐specific KASP assays in wheat (Allen *et al*., 2011) but also indicated that in the absence of software that would automatically annotate sequences with anchored bases, it was a time‐consuming process. The lack of availability of both the wild relative genome sequences and a complete wheat genome sequence made the approach taken in this study the most appropriate at the time. In future, automated pipelines such as PolyMarker™ (Ramirez‐Gonzalez *et al*., 2015) and MAGICBOX™ (Curry *et al*., 2016) will be very useful tools to redesign failed assays or design chromosome‐specific assays for newly discovered SNPs between wheat and its wild relatives.

### SNP validation and characterization

A subset of 1000 putative interspecific SNPs was selected for conversion into KASP assays (Tables 1 and S3). Genotyping results (Tables S4 and S5) showed that 73% of the SNPs were converted into a working KASP assay. This conversion rate is lower compared to another study in which 96% were successfully validated to be polymorphic between wheat varieties (Allen *et al*., 2013) but still relatively high for a complex polyploid such as wheat (Edwards *et al*., 2009). However, it was noted that approximately half the failed assays amplified the wheat varieties but not the target wild relative accessions (Data S1). This could be due to several reasons such as sequencing errors leading to false positives during SNP discovery, inefficient primer design for the wild relative allele and/or suboptimal PCR conditions during genotyping (all attributed to the complex polyploid nature of some of the wild relative species such as *Th. elongatum* [decaploid] and *Th. intermedium* [hexaploid]). Of the assays that worked, 17 were found to be polymorphic within wheat probably due to the presence of homoeologous sequences that were not detected in the sequence data but were amplified by the KASP primers.

Of the 710 KASP assays that were polymorphic between the four main hexaploid wheats, and the ten wild relative species, ~88% were validated (monomorphic for the wheat allele) across a combination of hexaploid and tetraploid wheat varieties (Table S5; Data S1), suggesting that these markers hold promise as broad tools in wheat breeding.

It was also important to ensure that the markers were evenly distributed across the whole genome of the wild relatives. Due to a lack of availability of high‐quality reference genomes with ordered pseudomolecules for the wild species used in this study (except for *T. urartu*; Ling *et al*., 2018), it was not possible to easily ascertain the distribution of the KASP markers in the wild relative species. Thus, we selected various wheat–wild relative introgressions lines from previous studies, where the Axiom^®^ Wheat‐Relative Genotyping Array had been used for genotyping and genetic mapping (Cseh *et al*., 2019; Grewal *et al*., 2018a,b; King *et al*., 2017, 2018). Inclusion of these lines from backcross generations, for each wild relative species, ensured that all linkage groups were being detected by the KASP markers.

Most of the working assays for a target chromosome were able to distinguish the heterozygous samples from the homozygous samples in a segregating population and provided a null call in the corresponding Chinese Spring nullisomic–tetrasomic line and were thus classified as chromosome‐specific (Figure 2a–e). From among the validated assays, 620 were chromosome‐specific (Table 2) and 90 were chromosome‐nonspecific; that is, they detected more than one homoeologous loci in wheat (Figure 2f–h). Various wild relative species had validated chromosome‐specific assays that also worked in other species (Table S6), with more than half shown to be working for at least 3 wild relative species (Data S2), thereby demonstrating the diverse applicability of these assays for various wheat–wild relative breeding programmes. BLASTN results showed that ~62% of these chromosome‐specific assays were derived from single‐copy regions of the wheat genome (Data S3). It is possible that such regions were either unique to only one progenitor genome or one or more copies could have been lost after polyploidization. BLASTX results showed that 275 (44%) of the chromosome‐specific assays were in protein‐coding regions with ~52% of these being single‐copy loci in wheat (Data S3). Previous studies have hypothesized that co‐dominant SNP assays are most likely to be in single‐copy genes of as yet unknown function in wheat. Where they are found to be in 3‐copy genes, it is likely to be in 3′ UTR regions that are more divergent than protein‐coding sequence (Allen *et al*., 2013). In our study, these single‐copy regions were found in both landraces such as Chinese Spring, and modern cultivars such as Paragon, Pavon 76 and Highbury. Thus, it is possible that these contigs represent genes that were lost before or during the domestication process. Previous reports have documented intra‐ or intervarietal heterogeneity and gene loss within elite or inbred lines of wheat (Tokatlidis *et al*., 2004; Winfield *et al*., 2012).

BLASTN results of the chromosome‐specific (Data S3) and the chromosome‐nonspecific (Data S4) SNP sequences allowed the visualization of the distribution of all the KASP markers (Figure 3), diagnostic for the ten wild relatives used in this work, and identification of regions where gaps exist and need to be filled in the future through more SNP discovery, KASP assay design and validation.

### Genotyping with chromosome‐specific markers

When the lines needed to be self‐fertilized to create stable introgression lines, the Axiom^®^ Wheat‐Relative Genotyping array was unable to effectively distinguish between heterozygotes and homozygotes. Moreover, genotyping with the array could not provide any information about which specific wheat chromosome had recombined with the wild relative species. The development of the chromosome‐specific KASP markers has, for the first time, allowed the identification of homozygous introgressions and the site of recombination in wheat.

Where the introgressed segment from a wild relative chromosome is orthologous with the wheat chromosomes, its presence is indicated by heterozygous calls for chromosome‐specific markers on homoeologous loci across all three subgenomes of wheat (Figure 4a). This is because the markers were designed to be polymorphic between the wild relative genome and each of the three subgenomes within a homoeologous group. However, when the recombinant segment is homozygous in wheat, the loss of wheat alleles (due to both copies of the wheat loci on one subgenome being replaced by wild relative loci) results in a homozygous wild relative call for the chromosome‐specific markers on the recombinant wheat chromosome (Figure 4b) and hence allows for the identification of the site of introgression.

False positives of homozygosity could be obtained if there is a deletion of both copies of a wheat subgenome from a homoeologous group, which is the same as the one into which the wild relative segment has been introgressed. Gaps in the marker distribution, particularly in the distal regions of chromosomes, might result in difficulty in distinguishing between a large recombinant segment and a whole chromosome introgression and also in the failure to detect small telomeric introgressions. Another point to note is that these markers were designed assuming overall macro‐synteny between the wheat subgenomes and the wild relative genomes. However, there are wild relative genomes with major rearrangements compared to the wheat genome, such as *S. cereale* (Devos *et al*., 1993; Li *et al*., 2013), in which case known rearrangements must be taken into account. For less well‐characterized wild relatives, it will still be possible to use the markers to identify the presence of wild relative segments and to distinguish between heterozygous and homozygous introgressions.

## Conclusion

This study has described the design, validation and implementation of chromosome‐specific KASP markers in wheat. A majority of these markers are based on single‐copy regions in the wheat genome but where there are homoeologous copies of the target SNP sequence, ‘primer anchoring’ was used to design chromosome‐specific assays. Thus, 620 chromosome‐specific KASP assays have been validated which allow the rapid identification of homozygous wild relative introgressions in a wheat background and their potential site of recombination within wheat. In addition to this, 90 chromosome‐nonspecific KASP markers were also identified which can be used for the detection of wild relative chromatin in introgression lines. Most of the developed assays can be used for detection of multiple wild relative species used in this study. Thus, there is potential for these markers to be used to detect the presence of various other wild relative species and, moreover, for the detection of wild relative introgressions in a durum background. As such, these KASP assays could be a highly valuable resource, which will be of considerable interest to wheat researchers and, in particular, the breeding community.

## Experimental procedures

### Plant material

Various tetraploid and hexaploid wheat varieties and different accessions of ten wild relatives (Table S4) were grown for leaf tissue collection and nucleic acid extraction. The whole set of Chinese Spring nullisomic–tetrasomic lines was obtained through the Germplasm Resource Unit (John Innes Centre; http://www.seedstor.ac.uk). The backcross populations, created from crossing each of the wild relatives with the wheat cv. Paragon, were generated at the Nottingham BBSRC Wheat Research Centre.

All plants were grown in pots in John Innes No. 2 soil and maintained in a glasshouse at 18–25 °C under 16‐h light and 8‐h dark conditions. Leaf tissues were harvested from 3‐week‐old plants. All harvested tissues were immediately frozen on liquid nitrogen and stored at −80 °C until nucleic acid extraction.

### Nucleic acid extraction

Genomic DNA was extracted according to the Somers and Chao protocol (http://maswheat.ucdavis.edu/PDF/DNA0003.pdf, verified 21 January 2019, original reference in Pallotta *et al*., 2003). For wild relatives with multiple accessions, the genomic DNA was pooled into one sample.

### Primer design

All SNP probe sequences on the Axiom^®^ Wheat‐Relative Genotyping Array were used in a BLASTN search (e‐value cut‐off of 1e‐05) against the wheat reference sequence (IWGSC CSS v3; IWGSC *et al*., 2014) to find probes that had a BLAST hit to only one contig in the wheat genome. Primers were designed from the flanking 500 bp sequence using Primer 3 v4.1.0 (Untergasser *et al*., 2012) with default primer size and Tm conditions. Primers were ordered through Eurofins Genomics, Germany.

### Polymerase chain reaction (PCR) and sequencing

All primers were used for PCR amplification of genomic DNA using a touchdown program on the Mastercycler nexus GSX1 (Eppendorf, Germany): 95 °C for 5 min, then 10 cycles of 95 °C for 1 min, 65 °C for 30 s [−1 °C per cycle] and 72 °C for 2 min, followed by 40 cycles of 95 °C for 1 min, 58 °C for 30 s and 72 °C for 2 min. The amplification products were run on a 1.5% agarose gel with size marker Hyperladder™ 1 kb (Bioline, UK). DNA bands (~ 500 bp) were cut from the gel, cleaned using the NucleoSpin Gel and PCR Clean‐up kit (Macherey‐Nagel, Düren, Germany) and sent for Sanger sequencing (Source Biosciences, Nottingham, UK).

### SNP discovery

Sequences were visualized using Chromas Lite v2.1.1 (Technelysium, Australia). All sequences from the same primer pair were aligned using GeneDoc v2.7. The Chinese Spring sequence, for each primer pair, was used in a BLASTN search (e‐value cut‐off of 1e‐05) against the new wheat reference sequence (IWGSC RefSeq v1; IWGSC *et al*., 2018) to check for homoeologous sequences and obtain the physical position of the target SNP. The target interspecific SNP was annotated by its IUPAC code and square brackets. Any other SNPs found in the 100‐bp region flanking the target SNP were also annotated with the corresponding IUPAC code. If a SNP‐containing sequence had more than one homoeologous copy in wheat, then any subgenome‐specific bases, for the target subgenome, in the 100‐bp sequence flanking the SNP were annotated with chevrons.

### KASP™ assay design and validation

For each putative SNP, KASP™ assays containing two allele‐specific forward primers and one common reverse primer (Data S5) were designed (LGC Biosearch Technologies, UK) using the annotated SNP sequences. Leaf tissues from all the ten backcross populations (Table S5), the parental wheat and wild relative accessions, different tetraploid and hexaploid wheat and the Chinese Spring nullisomic–tetrasomic lines were sent for DNA extraction and genotyping with the KASP™ assays (LGC Biosearch Technologies, Middlesex, UK).

### Multicolour genomic *in situ* hybridization (mcGISH)

Preparation of the root metaphase chromosome spreads, the protocol for the mcGISH and the image capture was as described in King *et al*. (2017). All slides were probed with labelled genomic DNA of the three putative diploid progenitors of bread wheat, that is *T. urartu* (A genome), *Ae. speltoides* (B genome) and *Ae. tauschii* (D genome). Additionally, introgression lines with segments from *Th. bessarabicum* and *Am. muticum* were probed with the respective wild relative's labelled genomic DNA. The genomic DNA of (i) *T. urartu* was labelled by nick translation with ChromaTide™ Alexa Fluor™ 488‐5‐dUTP (Invitrogen; C11397; coloured green), (ii) *Ae. speltoides* was labelled by nick translation with DEAC‐dUTP (Jena Bioscience; NU‐803‐DEAC; coloured blueish purple), (iii) *Ae. tauschii* was labelled with ChromaTide™ Alexa Fluor™ 594‐5‐dUTP (Invitrogen; C11400; coloured red), and (iv) *Th. bessarabicum* and *Am. muticum* were labelled by nick translation with ChromaTide™ Alexa Fluor™ 546‐14‐dUTP (Invitrogen; C11401; coloured yellow).

## Conflict of interest

On behalf of all authors, the corresponding author states that there is no conflict of interest.

## Author contribution

SG identified the wheat genome sequences to be amplified. SH‐E, UD, LB and JH designed the PCR primers, extracted the DNA and performed the PCR experiments for SNP discovery with assistance from CH. SG analysed sequenced data for SNP discovery with assistance from SH‐E. CY, SA and DS prepared leaf material to be sent LGC Biosearch Technologies. JY performed the KASP assay design and validation. SG performed the data analysis of the KASP genotyping project with assistance from PI. CY performed the GISH analysis. SG, IPK and JK conceived and designed the experiments. SG wrote the manuscript with assistance from JK and IPK. All authors read and approved the final manuscript.

## Supporting information


**Table S1** Distribution of the number of SNPs discovered for each wild relative species, on the wheat chromosomes.

**Table S2** Distribution of second BLASTN results, for the discovered SNPs, across each wheat chromosome.

**Table S3** Distribution of the number of SNPs selected for KASP assay design, for each wild relative species, on the wheat chromosomes.

**Table S4** Details of the parental lines used for SNP discovery and validation.

**Table S5** Number of lines in each backcross population, for a wild relative species, used for genotyping.

**Table S6** Number of chromosome‐specific KASP assays common between two wild relative species.

**Data S1** Genotypes of all the parental and control lines with working KASP assays.

**Data S2** Details of wild relative species validated for each polymorphic KASP assay.

**Data S3** BLASTN and BLASTX results for all chromosome‐specific SNPs.

**Data S4** BLASTN results for all chromosome‐nonspecific SNPs.

**Data S5** KASP assay primer details for all selected SNPs.
